# Atomistic scale investigation of cation ordering and phase stability in Cs-substituted Ba_1.33_Zn_1.33_Ti_6.67_O_16_, Ba_1.33_Ga_2.66_Ti_5.67_O_16_ and Ba_1.33_Al_2.66_Ti_5.33_O_16_ hollandite

**DOI:** 10.1038/s41598-018-22982-7

**Published:** 2018-03-22

**Authors:** Yi Wen, Yun Xu, Kyle S. Brinkman, Lindsay Shuller-Nickles

**Affiliations:** 1 Department of Environmental Engineering and Earth Sciences, Clemson University, Clemson, SC USA; 2 Department of Materials Science and Engineering, Clemson University, Clemson, SC USA; 3 Center for Nuclear Environmental Engineering and Science and Radioactive Waste Management (NEESRWM), Clemson University, Clemson, SC USA

## Abstract

The titanate-based hollandite structure is proposed as an effective ceramic waste form for Cs-immobilization. In this study, quantum-mechanical calculations were used to quantify the impact of A-site and B-site ordering on the structural stability of hollandite with compositions Ba_x_Cs_y_(M_z_Ti_8-z_)O_16_, where M = Zn^2+^, Ga^3+^, and Al^3+^. The calculated enthalpy of formation agrees with experimental measurements of related hollandite phases from melt solution calorimetry. Ground state geometry optimizations show that, for intermediate compositions (*e.g*., CsBaGa_6_Ti_18_O_48_), the presence of both Cs and Ba in the A-site tunnels is not energetically favored. However, the decay heat generated during storage of the Cs-containing waste form may overcome the energetics of Ba and Cs mixing in the tunnel structure of hollandite. The ability of the hollandite structure to accommodate the radioparagenesis of Cs to Ba is critical for long term performance of the waste. For the first time, B-site ordering was observed along the tunnel direction ([001] zone axis) for the Ga-hollandite compositions, as well as the intermediate Al-hollandite composition. These compositionally dependent structural features, and associated formation enthalpies, are of importance to the stability and radiation damage tolerance of ceramic waste forms.

## Introduction

Cesium is a key fission product in spent nuclear fuel. With a half-life of 30 years, Cs-137 releases a large amount of heat from radioactive decay. Current spent fuel and high level waste disposal strategies call for Cs-immobilization in glass waste forms; however, multiphase ceramic waste forms have the potential to sequester the myriad of elements included in advanced multi-component waste streams^1^. A Ba- and Cs-containing hollandite is predicted to form during fabrication of multiphase ceramics from melt processing^2^. Hollandite has a general formula of A_x_(Ti,B)_8_O_16_ and structurally consists of TiO_6_ and BO_6_ octahedra, with A-site cations held within the tunnel cavities^3^. Cs is known to incorporate into the A-site tunnels of hollandite during processing, while other dopants such as Zn and Ga are incorporated as B-site cations to maintain charge neutrality^4^. Since ^137^Ba is a major decay product of ^137^Cs, Ba-hollandite is a promising material to account for radioparagenesis.

The typical hollandite composition reported in waste form literature is close to Ba_1.04_Cs_0.24_Ga_2.32_Ti_5.68_O_16_ with 4 at% loading; however, recent work has investigated higher Cs-loading with the synthesis of a pure Cs-hollandite end member (Cs_1.33_Ga_1.33_Ti_6.67_O_16_; 21 at% loading)^5,6^. In terms of stability, different B-site cations may impact the thermo-stability of the hollandite structure^2^. Different B-site cations also impact the amount of A-site cations in the lattice necessary to maintain charge neutrality. A challenging stability problem arises in multiphase ceramic waste-forms containing more than one type of B-site cation. Studying the potential for cation and vacancy ordering in hollandite structures will contribute to the understanding of the stability of hollandite over geologic timescales associated with waste storage.

In our previous work, we investigated the compositional effect of the A site of Ga-doped hollandite, where increasing Cs concentration corresponds to increased structural and thermodynamic stability of the hollandite phase^5,6^. Costa *et al*.^7^ discussed B-site dopant effects on the thermodynamic stability of hollandite for a single composition of Ba_1.04_Cs_0.24_(M, Ti)_8_O_16_. In this paper, both the A-site composition and B-site dopant effects are systematically explored using density functional theory calculations.

Density functional theory (DFT) calculations have been used to investigate cation incorporation^8–12^ and defect ordering^13–15^ in ceramic waste form materials. Fluorite-related structures, such as pyrochlore, have proven resistant to radiation damage^16,17^, which has been attributed to the flexibility of the system to withstand disorder^18,19^. Such disorder has been observed experimentally and both confirmed and predicted computationally. Here, DFT calculations are used to quantitatively compare the energetic favorability of different A-site and B-site configurations across a range of hollandite compositions with a fixed A-site occupancy. Ordering observed through theoretical simulations can guide future experimental exploration into preferential cation ordering in hollandite with implications on radiation damage tolerance and long-term stability related to radioparagenesis.

## Methods

### Density functional theory calculations

Quantum-mechanical DFT calculations were performed using the generalized gradient approximation (GGA) with the Perdew-Burke-Ernzerhof functional^20^ for the electron correlation and exchange energy as implemented in the CAmbridge Serial Total Energy Package (CASTEP)^21^. Ultra-soft pseudopotentials were used to describe the behavior of the core electrons and their interactions with the valence electrons, while Ba 5s/4d^2^ 5p^6^ 6s^2^, Cs 5s/4d^2^ 5p^6^ 6s^1^, Zn 3d^10^ 4s^2^, Ga 3d^10^ 4s^2^ 4p^1^, Al 3s^2^ 3p^1^, Ti 3s^2^ 3p^6^ 3d^2^ 4s^2^, and O 2s^2^2p^4^ valence electrons were calculated explicitly. A k-point mesh of 2 × 2 × 2 (4 k-points total) was used for the 1 × 1 × 3 hollandite supercell calculations. A k-point mesh of 1 × 2 × 2 (2 k-points total) was used for the 2 × 1 × 3 supercells. An energy cut-off of 700 eV was chosen based on initial convergence testing. Electronic and geometry convergence criteria for total energy per atom was set to 2 × 10^−6^ eV and 2 × 10^−5^ eV, respectively.

### Hollandite compositional range

Hollandite is a titanate structure composed of octahedral B-site cations that form tunnels, which incorporate A-site cations. The A-site is composed of mono and/or divalent cations, specifically Cs^+^ and Ba^2+^. The occupancy of the A-site ranges from one-half to one mole fraction depending on both the A-site and B-site dopant^22^. Hollandite with divalent A-site cations generally have tunnel site occupancy near one-half and less than two-thirds, while hollandite with monovalent cations, such as Na, K or Cs, have tunnel site occupancy of two-thirds to three-quarters, depending on the cation size. For this study the occupancy was 1.33/2 (two-thirds filled), which is achievable for both divalent Ba and monovalent Cs. For the 1 × 1 × 3 supercell used in this study, 4 of the 6 A-sites are occupied. Additional calculations were performed for 2 × 1 × 3 supercells of a subset of hollandite compositions to clarify energetic and structural trends according to increasing Cs concentration. The B-site is predominately composed of Ti^4+^, as well as di and/or trivalent cations for charge-balance. For this study, Zn^2+^ and Ga^3+^ were used. Al-hollandite was also included for comparison to experimental calorimetry measurements^7^. Table 1 lists the 13 different compositions evaluated in the present work. A range of configurations were studied for the end-member (pure Ba and pure Cs-hollandite) and intermediate member compositions, leading to an understanding of cation ordering at both the A- and B-sites. The configurations evaluated during the current study are available from the corresponding author on reasonable request.

**Table 1:** End-member and intermediate member compositions studied. Compositions are normalized to a 1 × 1 × 1 unit cell.

Ba-end member	Intermediate member	Cs-end member
Ba_1.33_Zn_1.33_Ti_6.67_O_16_	Ba_0.67_Cs_0.67_Zn_1.00_Ti_7.00_O_16_	Cs_1.33_Zn_0.67_Ti_7.33_O_16_
Ba_1.33_Ga_2.67_Ti_5.33_O_16_	Ba_1.00_Cs_0.33_Ga_2.33_Ti_5.67_O_16_	Cs_1.33_Ga_1.33_Ti_6.67_O_16_
	Ba_0.67_Cs_0.67_Ga_2.00_Ti_6.00_O_16_	
	Ba_0.33_Cs_1.00_Ga_1.67_Ti_6.33_O_16_	
Ba_1.33_Al_2.67_Ti_5.33_O_16_	Ba_1.00_Cs_0.33_Al_2.33_Ti_5.67_O_16_	Cs_1.33_Al_1.33_Ti_6.67_O_16_
	Ba_0.67_Cs_0.67_Al_2.00_Ti_6.00_O_16_	
	Ba_0.33_Cs_1.00_Al_1.67_Ti_6.33_O_16_	

### Calculation of thermodynamic properties

The total energy from the DFT calculations does not include any contribution from entropy (either configurational or vibrational); therefore, the total energy can be considered an enthalpy. In fact, the total energy from the CASTEP calculations is a sum of the energies of the zero valent components. The total CASTEP energies are used only for comparison of different hollandite configurations with the same composition. To gleam translatable meaning from the calculated total energy, the enthalpy of formation based on the simple oxides and the enthalpy of mixing across a binary solid solution are computed^23^. In total, three different energetic terms are highlighted for the evaluation of ordering and stability in the hollandite system − 1. Change in total energy for systems with the same composition, but differing configuration (ΔE_conf_), 2. Enthalpy of formation for a given configuration based on formation from the simple oxides (ΔH^f^_oxides_), and 3. Enthalpy of mixing across a given binary solid solution series, where the end-member compositions are pure Ba-hollandite and pure Cs-hollandite (ΔH_mix_). For example, the formation enthalpy for the Ba-Zn end-member hollandite composition (Ba_4_Zn_4_Ti_20_O_48_) is calculated according to Equation 1. The enthalpy of formation indicates the thermodynamic likelihood that the composition will form a stable single phase hollandite.

1 $$\begin{array}{c}4{\rm{BaO}}+4{\rm{ZnO}}+20{{\rm{TiO}}}_{2}\to {{\rm{Ba}}}_{4}{{\rm{Zn}}}_{4}{{\rm{Ti}}}_{20}{{\rm{O}}}_{48}\\ {{\rm{\Delta }}H}_{{\rm{oxides}}}^{{\rm{f}}}={\rm{E}}({{\rm{Ba}}}_{4}{{\rm{Zn}}}_{4}{{\rm{Ti}}}_{20}{{\rm{O}}}_{48})-4\,\ast \,{\rm{E}}({\rm{BaO}})-4\,\ast \,{\rm{E}}({\rm{ZnO}})-20\,\ast \,{\rm{E}}({{\rm{TiO}}}_{2})\end{array}$$

The enthalpy of mixing for the solid solutions are determined based on a linear combination of the lowest enthalpy end member configurations, where a positive enthalpy of mixing value indicates a preference for a mechanical mixture of the end members^24^. The lowest enthalpy configurations for the end-members are set to zero, and the enthalpy for the other configurations are with respect to the lowest enthalpy configuration. The enthalpy for the intermediate composition is actually the enthalpy of mixing with respect to the lowest enthalpy end-member configurations.

Configurational entropy is considered in the final evaluation of cation ordering for hollandite compositions containing trivalent B-site cations. The mathematical expression for the configurational entropy for the hollandite system is complicated due to the coupled nature of the solid solution, which includes two-site mixing (*i.e*., mixing on both A and B sites) and an interdependency of the A-site and B-site composition. Following the derivation for the configurational entropy for the plagioclase [(Ca_1−x_Na_x_)(Al_2−x_Si_2+x_)O_8_] coupled-substitution solid solution^25^, the configurational entropy for the 1 × 1 × 1 unit cell of hollandite, with the number of mixing components *N* = 4, is defined in Equation 2.

2 $$\begin{array}{rcl}{\rm{\Delta }}{S}_{holl}^{conf} & = & -1.33R[\frac{x}{1.33}ln\frac{x}{1.33}+\frac{(1-x)}{1.33}\,\mathrm{ln}\,\frac{(1-x)}{1.33}]\\  &  & -8R[\frac{(2.67-x)}{8}\,\mathrm{ln}\,\frac{(2.67-x)}{8}+\frac{(5.33+x)}{8}\,\mathrm{ln}\,\frac{(5.33+x)}{8}]\end{array}$$

where $x=mole\,fraction\,of\frac{Cs}{Ba+Cs}$. The configurational entropy is divided into two sets of terms, the first describing the A-site mixing and the second describing the B-site mixing, where the total number of occupied A-site cations is 1.33 and the total number of B-site cations is 8. An additional term may be added to consider the ordering of the vacancy in the A-site, such that *N* = 5 and $x=mole\,fraction\,of\frac{Cs}{Ba+Cs+vancany}$. The vacancy concentration is fixed in this series of calculations; therefore, the vacancy ordering term simply increases the magnitude of the configurational entropy by a factor of 9.481 J/molK according to Equation 3, where the A-site occupancy is 2/3.

3 $$\begin{array}{rcl}{\rm{\Delta }}{S}_{holl}^{conf} & = & -2R[\frac{x}{2}ln\frac{x}{2}+\frac{(1-x)}{2}\,\mathrm{ln}\,\frac{(1-x)}{2}+\frac{0.67}{2}ln\frac{0.67}{2}]\\  &  & -8R[\frac{(2.67-x)}{8}\,\mathrm{ln}\,\frac{(2.67-x)}{8}+\frac{(5.33+x)}{8}\,\mathrm{ln}\,\frac{(5.33+x)}{8}]\end{array}$$

## Results and Discussion

### B-site ordering

**Figure 1:**
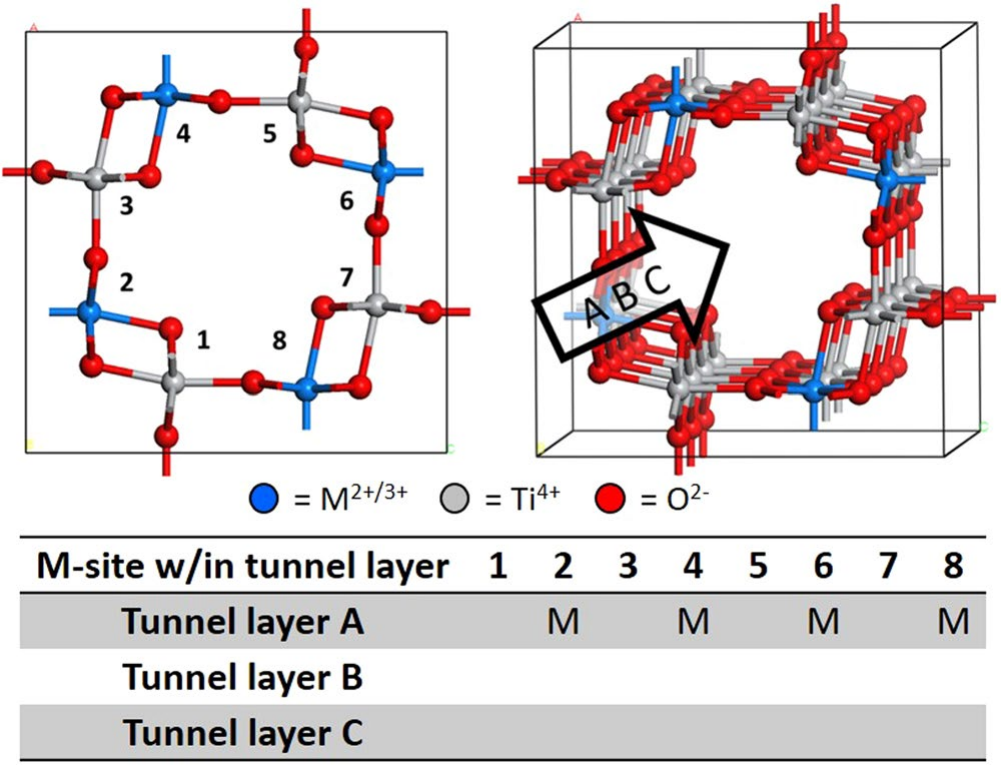
Schematic representing the B-site tunnel with the B-sites numbered within a tunnel layer, the tunnel layers labeled A, B, and C, and a configuration chart describing the B-site configuration. O (red), Ti (grey), M (blue).

B-site ordering was evaluated across the Ba-Cs solid solution with varying B-site dopants (Zn^2+^, Ga^3+^, and Al^3+^). Figure 1 is a schematic representing a single tunnel layer formed by the B-site cations, as well as the tunnel based on the stacking of the tunnel layers. The A-site cations are removed for clarity. Within a tunnel layer, the B-sites are numbered 1–8, while the tunnel layers within the 1 × 1 × 3 supercell are denoted A, B, and C. The configuration chart in Fig. 1 shows the general format used in describing the B-site configuration, where only the B-site dopants (*i.e*., non-Ti atoms) are noted.

**Figure 2:**
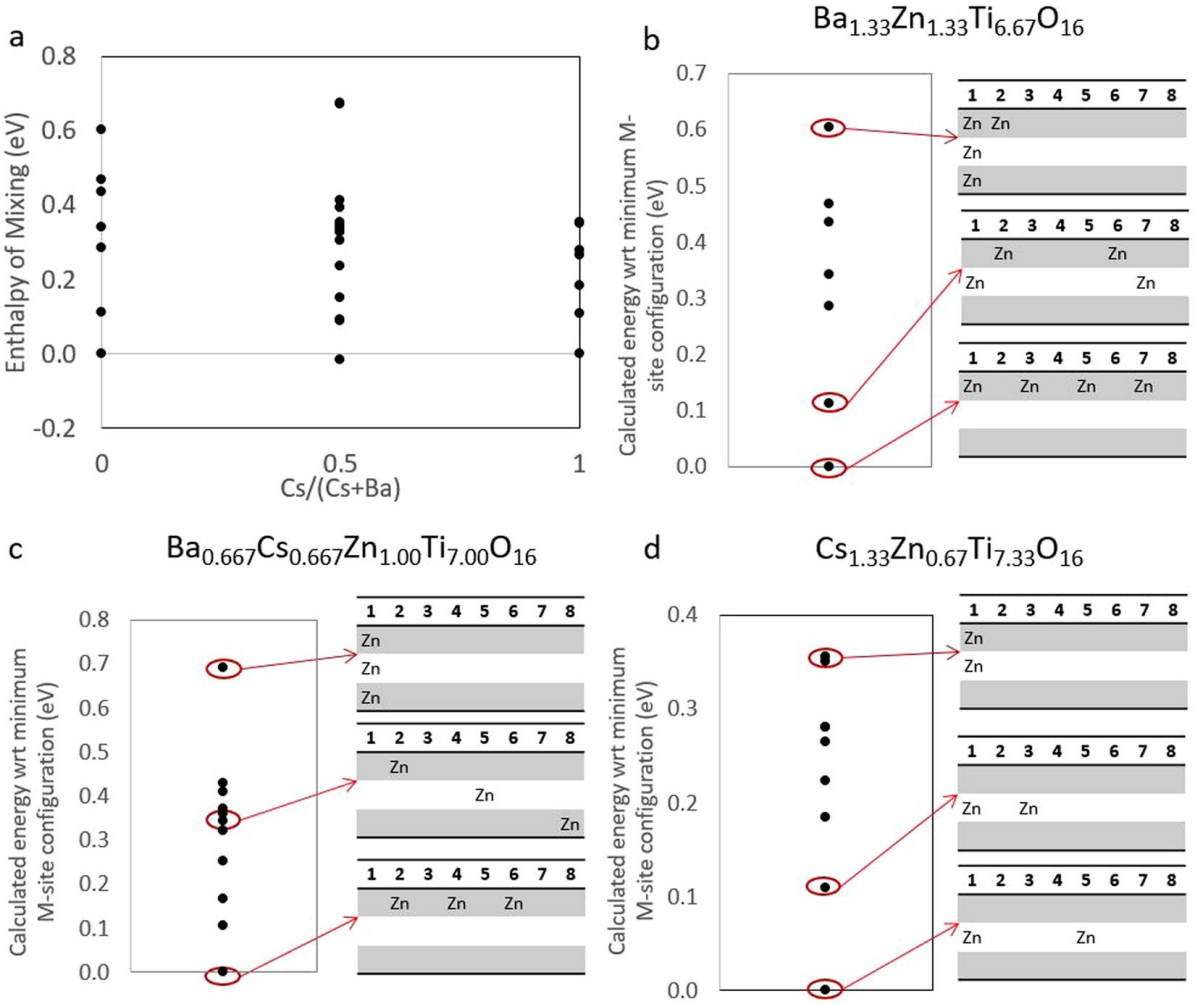
(**a**) The enthalpy of mixing curve highlights the intermediate composition (**c**) with respect to the end member compositions (**b**,**d**), where the x-axis is the concentration of Cs in the occupied A-sites. (**b**–**d**) Calculated energy with respect to (wrt) the minimum B-site configuration for a range of Zn-hollandite configurations, where the tables indicate the arrangement of B-site dopants within a tunnel layer (rows) or along the tunnel direction (columns). Note that the enthalpy of mixing energies (**a**) are the same as the energies reported in (**b**) and (**d**), but only similar to that reported in (**c**), where the minimum configuration for the energy comparison is the intermediate composition rather than the end-member composition(s).

Figure 2a shows the enthalpy of mixing for Zn-hollandite across the Ba-Cs solid solution, where the spread in the data for each composition shows the enthalpy range for varying B-site configurations. Vibrational and configurational entropy are not directly computed in these calculations; therefore, the enthalpy is equivalent to the total calculated energy. The range in energy associated with changing B-site configurations is detailed in Fig. 2b–d for Ba_1.33_Zn_1.33_Ti_6.67_O_16_, Ba_0.67_Cs_0.67_Zn_1.00_Ti_7.00_O_16_, and Cs_1.33_Zn_0.67_Ti_7.33_O_16_, respectively. Following the configuration chart described in Fig. 1, the lowest energy configuration, highest energy configuration, and an intermediate configuration are highlighted for each composition.

Generally, the lowest energy configuration is one in which the B-site dopants are arranged with the highest symmetry. For example, the Ba-end member for the Zn-hollandite, which contains four Zn atoms in the 1 × 1 × 3 supercell, has a lowest energy configuration in which the Zn are evenly spaced around the eight B-sites of a single tunnel layer (Fig. 2b). The Zn dopants prefer to order within a single tunnel layer (*e.g*., lowest energy configurations for all Zn-doped compositions tested in Fig. 2b–d) rather than along the tunnel direction (*e.g*., highest energy configurations for all Zn-doped compositions tested in Fig. 2b–d). Interestingly, Cs_1.33_Al_1.33_Ti_6.67_O_16_ prefers the same B-site configuration, where the B-site dopant is symmetrically spaced around one tunnel layer (*e.g*., Fig. 2b). Meanwhile, the Cs_1.33_Ga_1.33_Ti_6.67_O_16_ composition prefers have Ga ordered along the tunnel direction (*i.e*., the [001] zone axis). The difference in the B-site ordering for these systems stems from the difference in B-site dopant ionic radii, where the Ga^3+^ (R_Ga_ = 0.76 Å) is most similar to Ti^4+^ (R_Ti_ = 0.745 Å), while the Al^3+^ (R_Al_ = 0.675 Å) and Zn^2+^ (R_Zn_ = 0.88 Å) differ from the Ti^4+^ by 9% and 18%, respectively^26^.

**Figure 3:**
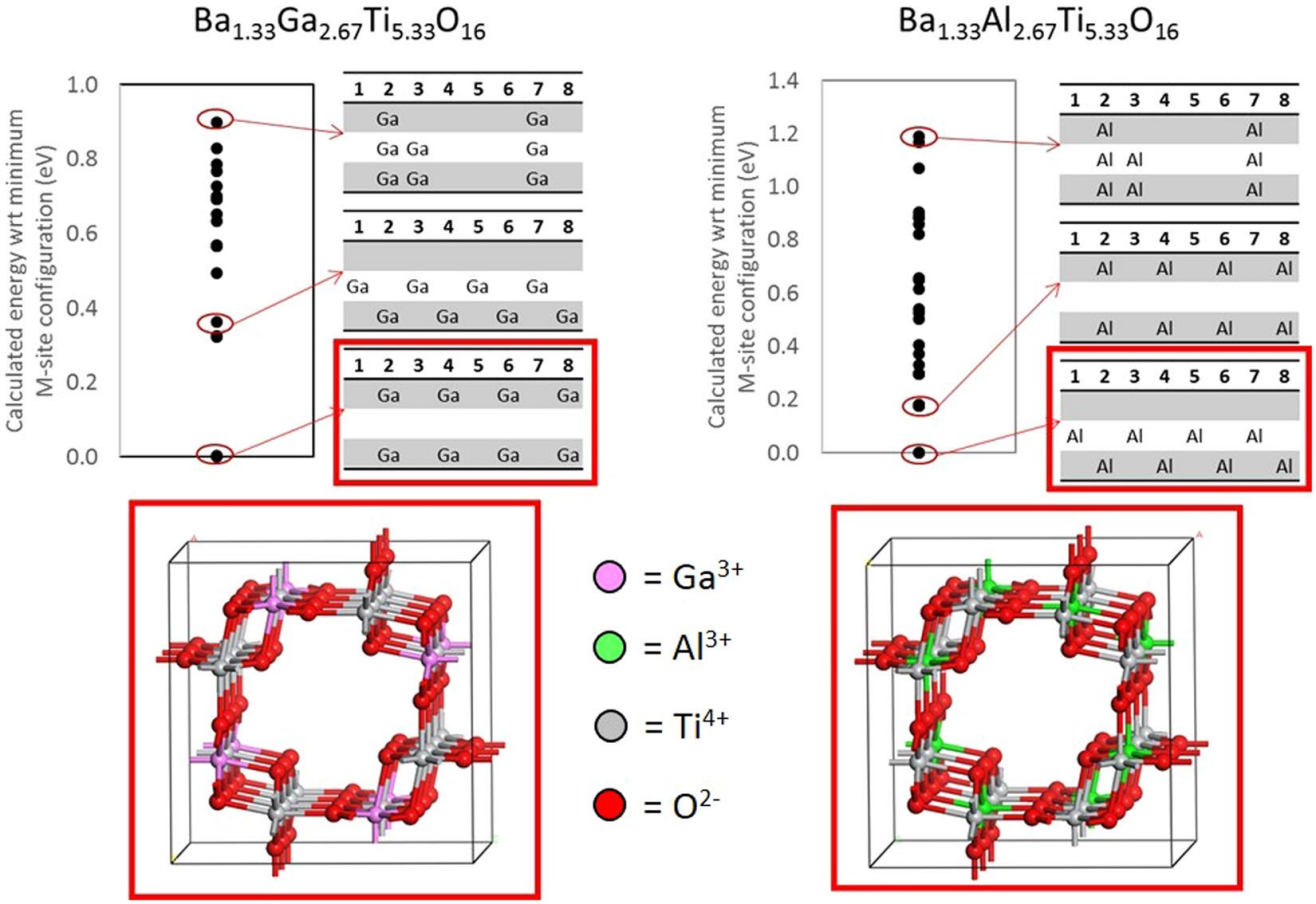
Range of energies for the Ba-end member compositions for Ga- and Al-hollandite with the configuration chart highlighted for the optimal configuration (lowest energy), an intermediate configuration, and the highest energy configuration. The ball and stick models show the lowest energy arrangement of the Ga (pink) and Al (green) B-site dopants. Note: Ti (grey), O (red), and A-site cations removed for clarity.

To maintain charge balance, the trivalent B-site dopants are incorporated with a higher concentration than the divalent B-site dopants. The increase in dopant concentration leads to changes in the favorability of the B-site configurations. Optimal configurations still maintain symmetry; however, some deviations are observed related to the size of the dopant cation. For example, for the Ba-end member (eight M^3+^ dopants), the lowest energy Ga-hollandite configuration prefers Ga dopants stacked in adjacent layers, while the Al-hollandite prefers the Al dopants to be shifted in adjacent layers (Fig. 3). The larger change in ionic radius between the Al^3+^ and Ti^4+^ (|∆R| = 0.07 Å), as compared with the Ga^3+^ and Ti^4+^ (|∆R| = 0.02 Å), likely causes the Al^3+^ in adjacent layers to be shifted.

The intermediate trivalent cation compositions contain six B-site dopants, and therefore, the symmetry is inherently broken. Six cations cannot be symmetrically spaced across eight sites. In these cases, the B-site dopants align along the tunnel direction, maintaining two-fold rotational symmetry, rather than the standard 4-fold rotation in the 4-dopant systems.

### A-site ordering

As expected, the A-site cations are distributed equally between tunnels within a unit cell; that is, for an occupancy of 1.33, two cations and one vacancy are located in each tunnel within the 1 × 1 × 3 and 2 × 1 × 3 supercells. For intermediate compositions, Cs and Ba prefer to arrange homogeneously within the tunnels of the 1 × 1 × 3 supercell, such that one tunnel contains two Cs atoms and the other contains two Ba atoms. For example, the intermediate composition Ba_2_Cs_2_Ga_6_Ti_18_O_48_ indicates that Ba and Cs prefer to be arranged homogeneously, such that each tunnel contains only one cation type; the homogenously ordered configuration is 0.0356 eV lower in energy than the configuration with A-site cations heterogeneously arranged. This energy difference is relatively low, as discussed below, and may be overcome with slightly elevated temperatures.

**Figure 4:**
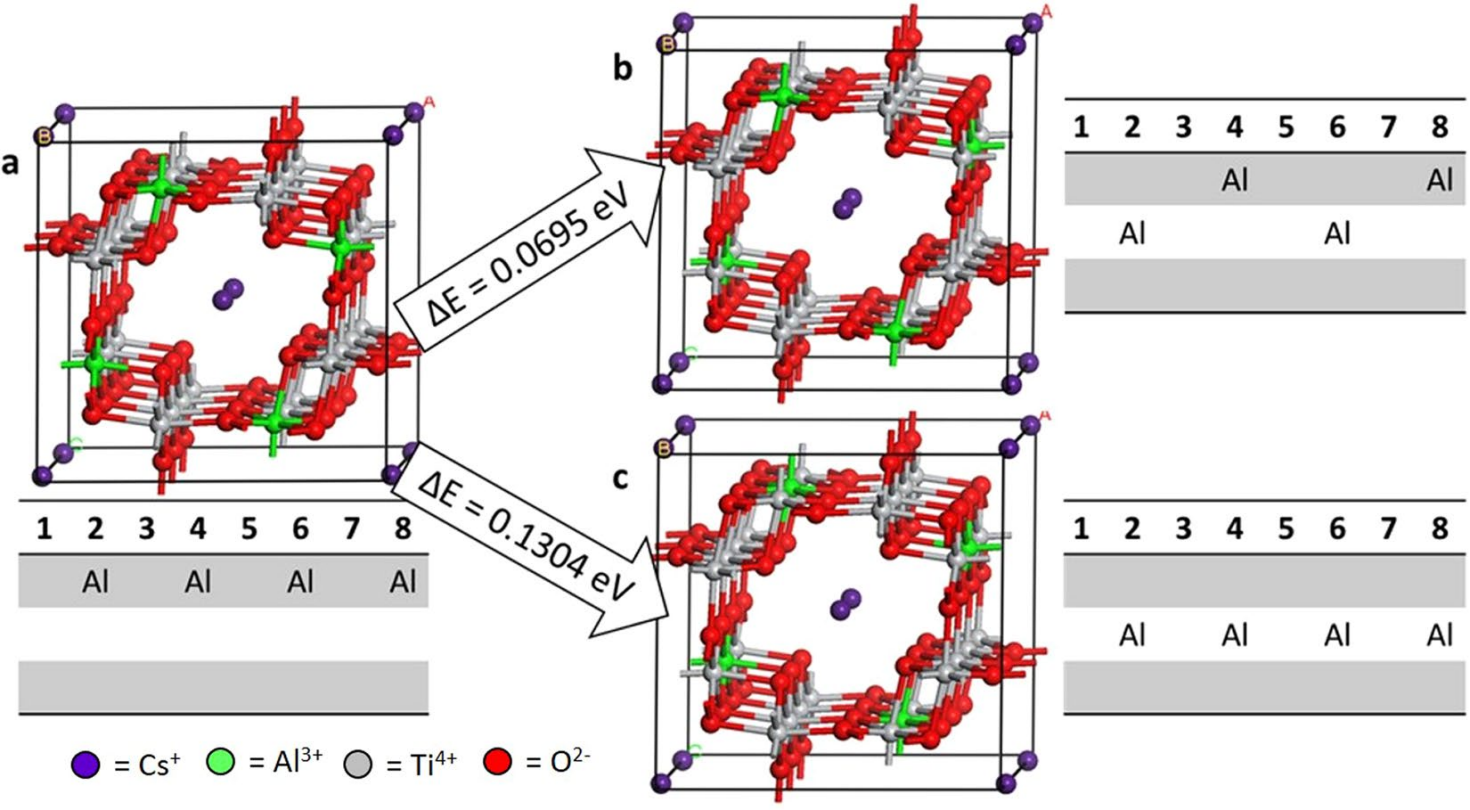
Impact of B-site ordering on the total energy of the Al-hollandite system demonstrated with ball-and-stick models, configuration charts, total energies, and change in energy with respect to the lowest energy configuration (**a**), where (**b**) shows the impact of a slight change in the B-site ordering and (**c**) shows the impact of the relative position of B-site and A-site cations. Cs (purple), Al (green), Ti (grey), O (red).

Cation mobility along the tunnel, and eventually out of the solid phase, is largely dependent on the distribution of the A-site cations and A-site vacancies, as well as the relationship between the A-site cations/vacancies and the B-site dopants. In general, cation-vacancy ordering on the A site is a function of site occupancy with higher occupied tunnels exhibiting no superlattice peaks in diffraction studies^27^. The minimum energy configuration for the CsAl-hollandite (Fig. 4a) has A-site ordering of Cs V Cs/V Cs Cs and a B-site ordering in which the four Al atoms are evenly spaced around a single tunnel layer. Shifting two Al such that the Al remain evenly spaced but span two tunnel layers results in an increase (less favorable) in energy of 0.0695 eV (Fig. 4b). More significantly, when the evenly spaced Al atoms are all located in a different tunnel layer (*i.e*., the middle layer rather than the outside layer) the total energy for the system increases by 0.1304 eV (Fig. 4c). This increase in energy with change in Al tunnel layer filling suggests that the relationship of the B-site substitutions with the A-site vacancy may play a noticeable role in changing the total energy of the system. The observation of the impact of vacancy ordering in the A-site on the overall system energy is supported when comparing different A-site ordering with the same B-site configuration. For example, for the case of Al evenly spaced in alternating tunnel layers (Fig. 4b), the A-site configuration Cs V Cs/V Cs Cs is favored over Cs Cs V/V Cs Cs by 0.0920 eV.

### Compositional controls on tunnel diameter

**Figure 5:**
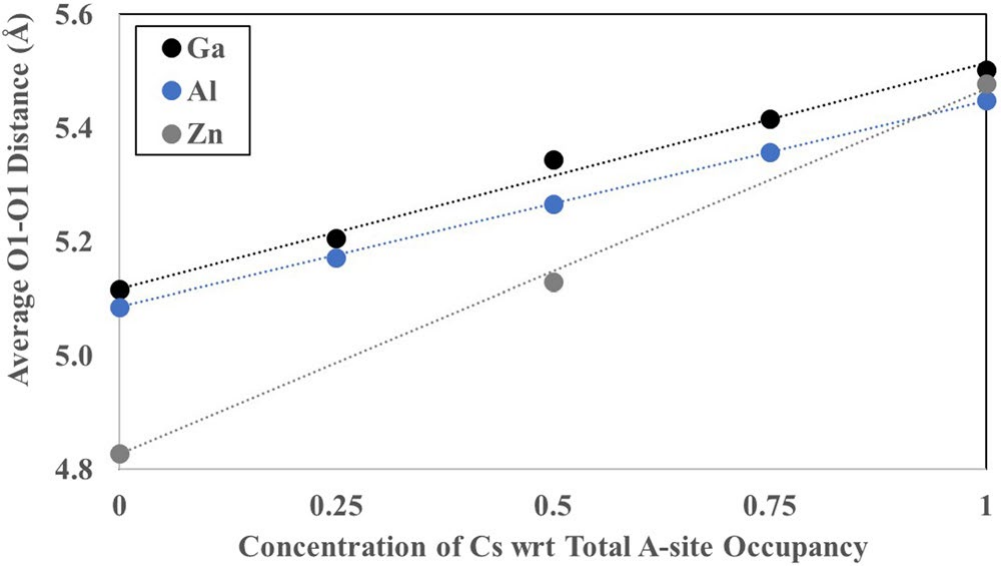
Average O1-O1 distance plotted against concentration of Cs for Ga- and Al -hollandite. Dotted lines are a linear fit to the data with a fixed intercept at the Ba-end member average O1-O1 distance.

The tunnel diameter, as described here by the diagonal O1-O1 distance, is a key factor that determines the mobility of tunnel cations^6^. The diagonal O1-O1 distance forms a bottleneck that inhibits cation mobility and is affected by both A-site and B-site composition. In general, larger A-site cations will expand the tunnel framework and increase the O1-O1 distance. The average O1-O1 distance increases with increasing Cs concentration (Fig. 5) because the number of tunnels containing Cs increases relative to the number of tunnels containing Ba. Both the ionic radius and oxidation state of the A-site cation contributes to the O1-O1 distance. The ionic radii of cubically-coordinated Cs^+^ and Ba^2+^ are 1.88 Å and 1.56 Å^26^, respectively. The lower formal charge of Cs (1+), as compared with Ba (2+), leads to longer Cs-O1 distances than Ba-O1 distances, and thus, longer overall O1-O1 distances across tunnels containing Cs. As noted from neutron diffraction studies, and confirmed with DFT calculations, the O1-O1 distance remains short enough to trap Cs in the hollandite tunnel, limiting cation diffusion^5^.

The oxidation state and ionic radius of B-site dopants also affect the O1-O1 distance. With increasing Cs^+^ concentration, fewer B-site dopants are required for charge balance, decreasing the impact of the B-site dopant on the O1-O1 distance. For example, the ionic radius of octahedrally-coordinated Zn^2+^ (0.880 Å) is 18% larger than Ti^4+^ (0.745 Å)^26^, but due to the large difference in oxidation state, only one Zn^2+^ dopant is needed for charge balance in the 1 × 1 × 3 supercell of the Cs-end member. Thus, the average O1-O1 distance for the Cs-end member of the Zn-hollandite, is similar to the Al- and Ga-hollandite (Fig. 5).

**Figure 6:**
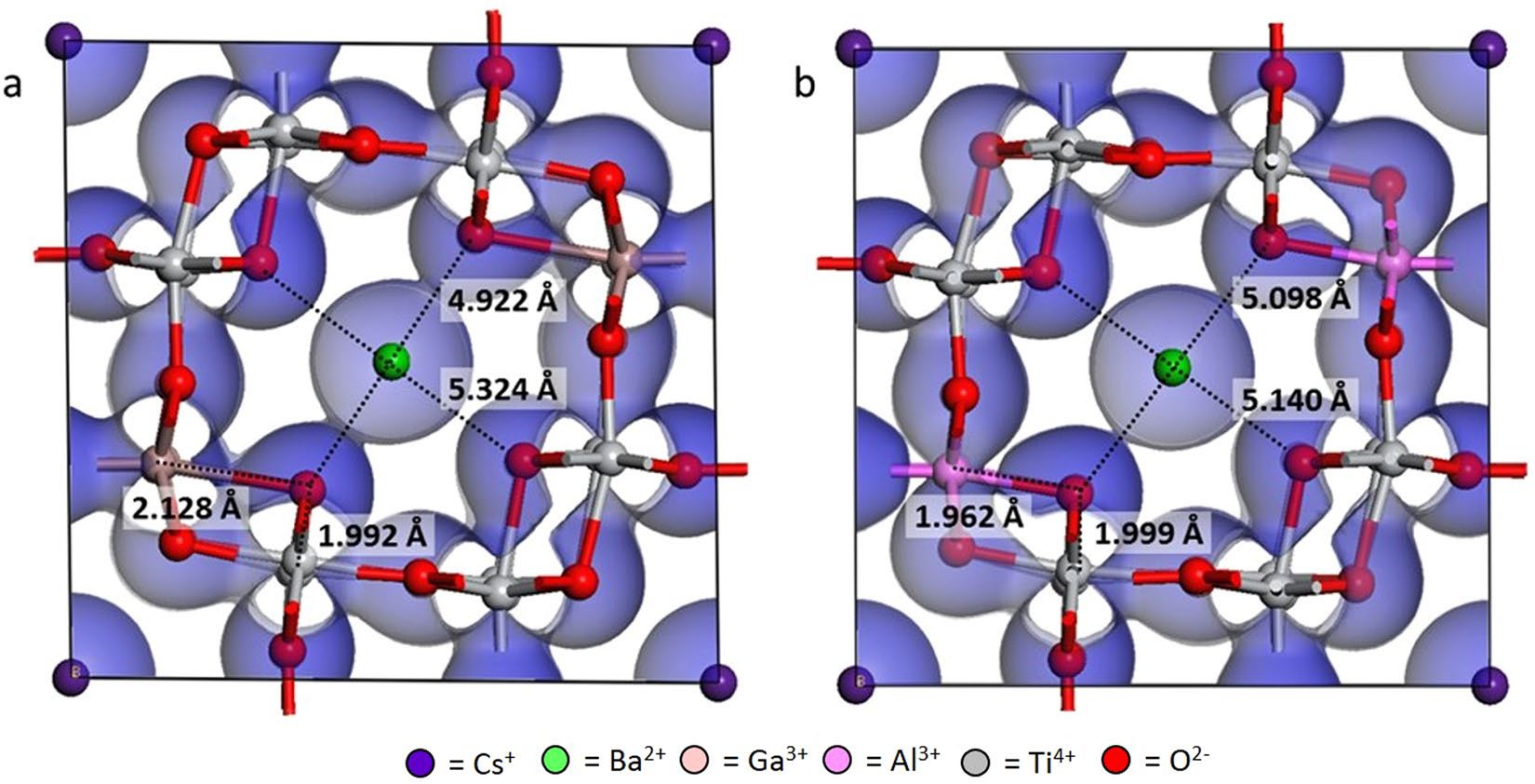
Ball and stick models of intermediate (**a**) Ga-hollandite and (**b**) Al-hollandite with the electron density projection overlaid in blue. Average bond distances are noted. Oxygen is red, Ti is grey, Ga is peach, Al is pink, Cs is purple, and Ba is green.

For cations with the same oxidation state (*i.e*., Ga^3+^ and Al^3+^), the impact of the B-site dopant on the O1-O1 distance is limited, yet consistent. While the difference in the average O1-O1 distance between the Ba- and Cs-end members of the Ga-hollandite solid solution is 0.386 Å, the difference between the Ga- and Al-hollandite for any A-site composition is, at most, 0.078 Å. Table 2 shows the average O1-O1 distances for O1 atoms bonded to two Ti atoms (O1-M-O1_Ti/Ti_), as well as to one Ti and one B-site dopant (O1-M-O1_Ti/B_), for the lowest energy configuration for the 50% Cs composition. The differing trends in the M-O1 vs. Ti-O1 distances for the Al- and Ga-hollandite are due to the size of the ionic radius of the B-site dopant, where the smaller Al dopant leads to shorter M-O1 distances, and in turn, a longer average O1-O1_Ti/M_ distance. The structure of the Ga-hollandite is more distorted than the Al-hollandite, as seen by the difference in the O1-O1 distances across the Ba-containing tunnel (Fig. 6). Noteably, the O1-O1 distance between O1 bonded to both Ga and Ti are shorter than the O1 bonded to only Ti. The same effect is not observed for the Al-hollandite intermediate system. Therefore, while the A-site cation (Cs vs. Ba) is the dominant control on the symmetry and stability of the hollandite tunnel structure, the B-site cation contributes to localized deviations in symmetry.

**Table 2:** Average M-O1 and O1-O1 distances for the lowest energy intermediate configurations for Ga- and Al-hollandite.

B-site cation	Ionic radius	M-O1	Ti-O1	O1-Cs-O1_Ti/M_	O1-Cs-O1_Ti/Ti_	O1-Ba-O1_Ti/M_	O1-Ba-O1_Ti/Ti_
Ga^3+^	0.760 Å	2.128	1.992	5.561	5.572	4.922	5.324
Al^3+^	0.675 Å	1.962	1.999	5.428	5.403	5.098	5.140

Note: The ionic radius of Ti^4+^ is 0.745 Å.

### Enthalpy of formation

**Figure 7:**
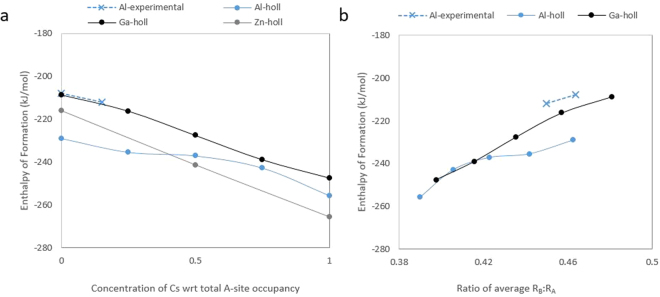
Enthalpy of formation (in kJ/mol) across Ba/Cs-hollandite solid solution *versus* (**a**) the Cs concentration with respect to total A-site occupancy and (**b**) the radius ration of average B-site to A-site cations (R_B_:R_A_). Experimental data is from Costa *et al*. 2013^7^ for composition Ba_1.24_Al_2.48_Ti_5.52_O_16_ and Xu *et al*. 2015^29^ for composition Ba_1.18_Cs_0.21_Al_2.44_Ti_5.53_O_16_.

The enthalpy of formation based on the oxide components was calculated for the lowest energy configuration for all compositions studied and compared with available calorimetry measurements for similar compositions (Fig. 7). Early calculations on the enthalpy of formation for Ga-hollandite^5^ are refined in this study with improved computational parameterization, particularly for the simple oxide phases, and increased number of cation configurations evaluated. The calculated enthalpy of formation for Ba_1.33_Al_2.66_Ti_5.44_O_16_ is −239.55 kJ/mol, while the measured enthalpy of formation for Ba_1.24_Al_2.48_Ti_5.52_O_16_ is −207.80 kJ/mol. The difference between the calculated and measured enthalpy of formation is about 15%, which is in good agreement considering the difference in composition and complexity of the crystalline system. Similar differences have been observed between calculated and measured oxides in literature. For example, the calculated and measured enthalpy of formation for metal uranates differ by about 10–40%, where limitations due to the computational theory are noted only for the system with 40% deviation from measurements^11,28^. Further, the calculated enthalpy of formation for the Ba-Al-hollandite end member ranged from −200.75 kJ/mol to −239.55 kJ/mol depending on the A-site and B-site configuration.

The enthalpy of formation decreases with increasing Cs concentration, where the total system charge is balanced by increasing the concentration of B-site dopants. Therefore, with higher Cs concentration, the deviation of the average B-site radius from 0.745 Å (ionic radius for octahedral Ti^4+^) increases. For systems with different types of B-site dopants, one might expect the enthalpy of formation to increase (become less favorable) with a greater difference in ionic radii between M and Ti^4+^. Further, the concentration of M-substitutions on the B-site contributes to the overall lattice strain and ultimately impacts the enthalpy of formation. The percent change between the M and Ti^4+^ ionic radii is 2%, 9%, and 18% for Ga^3+^, Al^3+^, and Zn^2+^, respectively; however, the enthalpy of formation is highest for Ga-hollandite and lowest for Zn-hollandite. This is likely due to the B-site valance, where the Zn-hollandite system requires fewer B-site substitutions to maintain charge neutrality.

Additional supercell calculations for the trivalent hollandite systems (Ga- and Al-hollandite) were performed to capture intermediate compositions of 0.25 and 0.75 mole fraction of Cs; thus, confirming the trend of decreasing enthalpy of formation with increasing Cs content. Figure 7b highlights the divergence of the enthalpy of formation trend due to the radio of average B-site and A-site radii, where the enthalpy of formation according to the ratio of average R_B_:R_A_ for both Ga- and Al-hollandite are the same if the average R_B_:R_A_ is ≤0.42. With higher average R_B_:R_A_, the enthalpy of formation for the Al-hollandite is lower than the Ga-hollandite.

### Implications for Long-term Disposal of Nuclear Waste

The B-site dopant has been noted for impacting the tunnel diameter^7^, where the lattice parameter increased with increasing average B-site ionic radius. The B-site ordering has the largest impact on structural stability of the system, where the energy difference between the most favorable and least favorable B-site configuration for the Cs-hollandite end members range between 0.26 eV and 0.36 eV. The propensity for B-site ordering also impacts the expected radiation damage tolerance of the material. Structures that are initially ordered, but have the ability to accommodate lattice disorder are known to possess larger radiation stability^17,19^. Limited studies are available on the radiation damage behavior of hollandite, however ordered B-site structures are expected to be more resistant to damage^29,30^.

Recently published neutron diffraction data for hollandite with high Cs-loading shows that the Cs concentration has a larger effect on the tunnel diameter and overall system symmetry than the B-site dopant type^5^. The calculated enthalpy of formation supports the conclusion that Cs stabilizes the formation of the hollandite system. Overall, the calculated variance of the enthalpy across the Ba-Cs solid solution decreases with increasing Cs concentration, further indicating the stabilization of the hollandite structure due to Cs in the tunnel cavity. The stabilization of the hollandite structure with increased Cs concentrations has positive implications for Cs-waste loading in hollandite. Thermodynamic comparisons with competitive Cs-host phases is necessary to quantify the expected maximum concentration of Cs in hollandite formed in a multi-phase ceramic.

Over time, ^137^Cs undergoes beta decay (τ_½_ = 30.08 yrs) to become ^137^Ba; therefore, understanding the arrangement of Cs and Ba on the A-site provides insight into the long term stability of hollandite due to the radioparagenesis of the waste components^31^. Ba and Cs prefer to segregate into different tunnels by about 0.036 eV. The implication of this energetic favorability can be estimated using a Boltzmann conversion of energy to temperature (E = kT, where E is energy, k is the Boltzmann constant of 8.617 × 10^−5^ eV/K, and T is temperature in K), where 0.036 eV equates to about 418 K (145 °C). While elevated, this temperature is far below the temperature used during waste form processing (1000–1500 °C)^2,32,33^. Commercially-derived high-level waste could generate enough self-heat from decay of fission products to result in an initial storage temperature as high as 600 °C. Even 100 years after emplacement, the waste temperature may be as high as 300 °C^34^. Therefore, enough energy may be supplied by the elevated temperature such that, upon decay, Ba may remain stable in the Cs-dominant tunnel of hollandite. Table 3 highlights the quantum-mechanically determined energy for different structural conditions examined in this work and the associated temperature based on the aforementioned Boltzmann conversion. Waste processing and storage temperature ranges are included for reference.

**Table 3:** Key structural conditions explored in quantum-mechanical calculations, calculated energy difference between most favorable and next most favorable configuration, and temperature (from Boltzman conversion) necessary to overcome the enthalpic favorability for given cation arrangement. Waste processing and storage temperature ranges are included for reference.

**Condition**	**Enthalpic favorability**	**Temperature** ^**+**^
Cs/Ba mixing in tunnel	0.036 eV (3.47 kJ/mol)	145 °C
Vacancy ordering	0.091 eV (8.78 kJ/mol)	800 °C
B-site ordering (Cs end-member)	0.26 eV–0.36 eV (25.1 kJ/mol–34.7 kJ/mol)	2750–3900 °C
Waste processing	—	1000–1500 °C
Initial commercial HLW storage	—	600 °C
100 years after waste emplacement	—	300 °C

^+^Temperature for the different cation configuration scenarios (rows 1–3) are calculated based on a Boltzman relationship of T and energy.

The smallest energetic difference for A-site vacancy ordering is 0.091 eV (~780 °C), which is associated with A-site ordering for the Cs-Al hollandite system. This energy difference is large enough to have implications for A-site cation mobility since it falls above the temperature range expected for storage of these waste forms. The occupancy explored in this study (1.33/2) is slightly higher than previous experimental work (1.24/2) in order to compare detailed computational geometry optimizations with experimental structural and mobility data. Thus, further analysis of A-site occupancy is critical to fully understand the cation mobility advantages of Cs-doped hollandite recently indicated^6^. The ordering of A-site (tunnel) cations has been both predicted^35^ and confirmed computationally and experimentally^27,36–38^.

**Figure 8:**
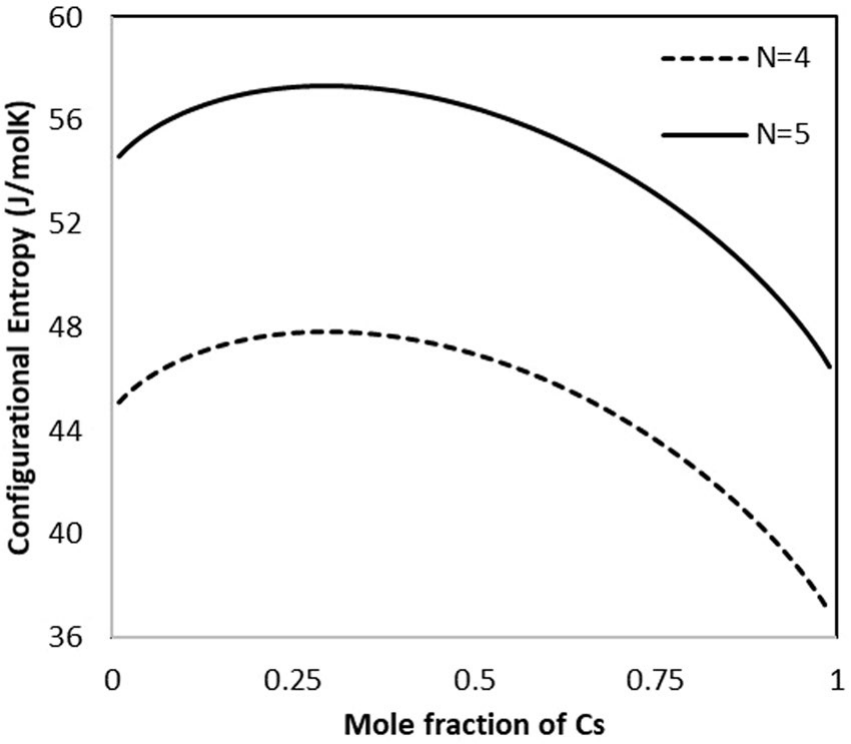
Configurational entropy for M^3+^-hollandite with respect to the mole fraction of Cs. N = 4 does not include ordering of the A-site vacancy, while N = 5 does.

The entropic contributions to thermodynamic stability were evaluated based on Equations 2 and 3. Figure 8 shows that the highest configurational entropy (57.3 J/mol K for N = 5 system) is associated with 0.296 mole fraction of Cs due to the coupling of the A-site and B-site mixing in this system. Further consideration of the vacancy ordering does not change the coupling of the A-site and B-site mixing for fixed occupancy systems. Thus, the N = 5 curve results in an increase in the magnitude of the configurational entropy associated with the additional mixing options on the A-site. The enthalpic favorability of B-site ordering and vacancy ordering are on the order of magnitude of the configurational entropy, particularly in the temperature range of waste form storage (below 600 °C); thus, the ordering in these systems can contribute to the overall stability of the ceramic waste form.

This study is the first to predict B-site (framework) ordering in Cs-loaded hollandite phases. The models presented here can guide future experimental efforts to confirm Ga ordering along the [001] zone axis (*i.e*., the tunnel direction). Following the trends in ionic radii, B-site dopants with ionic radii close to octahedral Ti^4+^ (0.745 Å) may also order along the tunnel direction. Further, the present work shows the coupling of B-site and A-site ordering, which, in turn, impacts the mobility of the A-site cations. Ultimately, quantification of the A-site cation mobility is critical for predicting long term potential for radionuclide release.

## References

[1] Ringwood AE (1985). Disposal of High-Level Nuclear Wastes - a Geological Perspective. Mineral Mag.

[2] Amoroso J, Marra J, Conradson SD, Tang M, Brinkman K (2014). Melt processed single phase hollandite waste forms for nuclear waste immobilization: Ba(1.0)Cs(0.3)A(2.3)Ti(5.7)O(16); A = Cr, Fe, Al. J Alloy Compd.

[3] Bystrom A, Bystrom AM (1949). Crystal Structure of Hollandite. Nature.

[4] Aubin-Chevaldonnet V (2007). Preparation and characterization of (Ba,Cs)(M,Ti)(8)O-16 (M = Al3+, Fe3+, Ga3+, Cr3+, Sc3+, Mg2+) hollandite ceramics developed for radioactive cesium immmobilization. J Nucl Mater.

[5] Xu, Y., Feygenson, M., Page, K., Shuller-Nickles, L. & Brinkman, K. S. Structural Evolution in Hollandite Solid Solutions Across the A-site Compositional Range. *Journal of the American Ceramic Society* (2016).

[6] Xu, Y. *et al*. A-site compositional effects in Ga-doped hollandite materials of the form BaxCsyGa2x + yTi8-2x-yO16: implications for Cs immobilization in crystalline ceramic waste forms. *Sci Rep* **6** (2016). 10.1038/srep27412 PMC4895209 27273791

[7] Costa GCC, Xu H, Navrotsky A (2013). Thermochemistry of Barium Hollandites. Journal of the American Ceramic Society.

[8] Zhao FA, Xiao HY, Jiang M, Liu ZJ, Zu XT (2015). A DFT plus U study of Pu immobilization in Gd2Zr2O7. J Nucl Mater.

[9] Nyman BJ, Bjorketun ME, Wahnstrom G (2011). Substitutional doping and oxygen vacancies in La2Zr2O7 pyrochlore oxide. Solid State Ionics.

[10] Rak, Z., Ewing, R. C. & Becker, U. Role of iron in the incorporation of uranium in ferric garnet matrices. *Phys Rev B* **84** (2011).

[11] Guo XF (2014). Cerium Substitution in Yittrium Iron Garnet: Valence State, Structure, and Energetics. Chemistry of Materials.

[12] Guo X (2014). Thermodynamics of thorium substitution in yttrium iron garnet: comparison of experimental and theoretical results. J Mater Chem A.

[13] Wang, J. W., Ewing, R. C. & Becker, U. Defect formation energy in pyrochlore: the effect of crystal size. *Materials Research Express* **1** (2014).

[14] Reader SW (2009). Cation disorder in pyrochlore ceramics: Y-89 MAS NMR and First-Principles Calculations. J Phys Chem C.

[15] Zhang J (2010). Intrinsic Structural Disorder and Radiation Response of Nanocrystalline Gd2(Ti0.65Zr0.35)2O7 Pyrochlore. J Phys Chem C.

[16] Ewing RC, Weber WJ, Lian J (2004). Nuclear waste disposal-pyrochlore (A(2)B(2)O(7)): Nuclear waste form for the immobilization of plutonium and “minor” actinides. Journal of Applied Physics.

[17] Weber WJ (1998). Radiation effects in crystalline ceramics for the immobilization of high-level nuclear waste and plutonium. J Mater Res.

[18] Levy MR, Grimes RW, Sickafus KE (2004). Disorder processes in A(3+)B(3+)O(3) compounds: implications for radiation tolerance. Philos. Mag..

[19] Sickafus KE (2007). Radiation-induced amorphization resistance and radiation tolerance in structurally related oxides. Nat Mater.

[20] Perdew JP, Burke K, Ernzerhof M (1996). Generalized gradient approximation made simple. Phys Rev Lett.

[21] Segall MD (2002). First-principles simulation: ideas, illustrations and the CASTEP code. J Phys-Condens Mat.

[22] Post JE, Burnham CW (1986). Modeling tunnel-cation displacements in hollandites using structure-energy calculations. Am Mineral.

[23] Shuller LC, Ewing RC, Becker U (2013). Np-incorporation into uranyl phases: A quantum-mechanical evaluation. J Nucl Mater.

[24] Shuller LC, Ewing RC, Becker U (2011). Thermodynamic properties of ThxU1-xO2 (0<x<1) based on quantum-mechanical calculations and Monte-Carlo simulations. J Nucl Mater.

[25] Saxena, S. K. *Thermodynamics of rock-forming crystalline solutions*. (Springer-Verlag 1973).

[26] Shannon RD (1976). Revised Effective Ionic-Radii and Systematic Studies of Interatomic Distances in Halides and Chalcogenides. Acta Crystallographica Section A.

[27] Torardi CC (1985). Synthesis and crystal structure of BaRu6O12: An ordered stoichiometric hollandite. Mater Res Bull.

[28] Guo XF (2016). U(V) in metal uranates: a combined experimental and theoretical study of MgUO4, CrUO4, and FeUO4. Dalton T.

[29] Tang M (2016). Heavy ion irradiations on synthetic hollandite-type materials: Ba(1.0)Cs(0.3)A(2.3)Ti(5.7)O(16) (A = Cr, Fe, Al). J Solid State Chem.

[30] Abdelouas A (2008). Effects of ionizing radiation on the hollandite structure-type: Ba0.85Cs0.26Al1.35Fe0.77Ti5.90O16. Am Mineral.

[31] Jiang C (2010). Using “radioparagenesis” to design robust nuclear waste forms. Energ Environ Sci.

[32] Crum, J. V. *et al*. Baseline Glass Development for Combined Fission Products Waste Streams. (Pacific Northwest NationalLaboratory, Richland, WA 2009).

[33] Crum JV, Turo L, Riley B, Tang M, Kossoy A (2012). Multi-Phase Glass-Ceramics as a Waste Form for Combined Fission Products: Alkalis, Alkaline Earths, Lanthanides, and Transition Metals. Journal of the American Ceramic Society.

[34] Ewing RC, Weber WJ, Clinard FW (1995). Radiation Effects in Nuclear Waste Forms for High Level Radioactive Waste. Progress in Nuclear Energy.

[35] Dryden JS, Wadsley AD (1958). The Structure and Dielectric Properties of Compounds with the Formula Ba_X_(Ti_8-X_Mg_X_)O_16_. Transactions of the Faraday Society.

[36] Pring A, Jefferson DA (1983). Incommensurate Super-Lattice Ordering in Priderite. Mineral Mag.

[37] Szymanski JT (1986). The Crystal-Structure of Mannardite, a New Hydrated Cryptomelane-Group (Hollandite) Mineral with a Doubled Short Axis. Canadian Mineralogist.

[38] Pring A, Smith DJ, Jefferson DA (1983). Supercell ordering in a hollandite-type phase: Potassium magnesium antimony oxide. J Solid State Chem.

